# Characteristic Metabolism of Free Amino Acids in Cetacean Plasma: Cluster Analysis and Comparison with Mice

**DOI:** 10.1371/journal.pone.0013808

**Published:** 2010-11-02

**Authors:** Kazuki Miyaji, Kenji Nagao, Makoto Bannai, Hiroshi Asakawa, Kaoru Kohyama, Dai Ohtsu, Fumio Terasawa, Shu Ito, Hajime Iwao, Nobuyo Ohtani, Mitsuaki Ohta

**Affiliations:** 1 Department of Animal Science and Biotechnology, Azabu University, Sagamihara, Japan; 2 Institute of Life Sciences, Ajinomoto Co. Inc., Kawasaki, Japan; 3 Department of Animal Care, Shimoda Floating Aquarium, Shimoda, Japan; 4 Department of Animal Care and Management, Izu-Mito Sea Paradise, Numazu, Japan; 5 Aqua Resorts, Yokohama-Hakkeijima Sea Paradise, Yokohama, Japan; 6 Enoshima Aquarium, Fujisawa, Japan; 7 Veterinary Hospital, Adventure World, Nishimuro, Japan; 8 Exhibition Division, Niigata City Aquarium Marinepia Nihonkai, Niigata, Japan; Universidad Europea de Madrid, Spain

## Abstract

From an evolutionary perspective, the ancestors of cetaceans first lived in terrestrial environments prior to adapting to aquatic environments. Whereas anatomical and morphological adaptations to aquatic environments have been well studied, few studies have focused on physiological changes. We focused on plasma amino acid concentrations (aminograms) since they show distinct patterns under various physiological conditions. Plasma and urine aminograms were obtained from bottlenose dolphins, pacific white-sided dolphins, Risso's dolphins, false-killer whales and C57BL/6J and ICR mice. Hierarchical cluster analyses were employed to uncover a multitude of amino acid relationships among different species, which can help us understand the complex interrelations comprising metabolic adaptations. The cetacean aminograms formed a cluster that was markedly distinguishable from the mouse cluster, indicating that cetaceans and terrestrial mammals have quite different metabolic machinery for amino acids. Levels of carnosine and 3-methylhistidine, both of which are antioxidants, were substantially higher in cetaceans. Urea was markedly elevated in cetaceans, whereas the level of urea cycle-related amino acids was lower. Because diving mammals must cope with high rates of reactive oxygen species generation due to alterations in apnea/reoxygenation and ischemia-reperfusion processes, high concentrations of antioxidative amino acids are advantageous. Moreover, shifting the set point of urea cycle may be an adaption used for body water conservation in the hyperosmotic sea water environment, because urea functions as a major blood osmolyte. Furthermore, since dolphins are kept in many aquariums for observation, the evaluation of these aminograms may provide useful diagnostic indices for the assessment of cetacean health in artificial environments in the future.

## Introduction

From an evolutionary perspective, the ancestors of cetaceans first lived in a terrestrial environment prior to adapting to an aquatic environment. The environmental changes that these cetacean ancestors encountered, spending their lifetime entirely underwater, were likely drastic, resulting in strong selection pressures. Anatomical and morphological adaptations to living underwater, such as a streamlined body shape and the location of the blowhole, have been well studied [1], [2]. In addition, certain adaptive physiological properties, such as a higher basal metabolic rate and a lower maximum rate of oxygen consumption [3], [4], are illustrated in cetaceans. There are, however, further unanswered questions to be investigated concerning the physiological changes that occurred over the course of evolution as a result of adaptation to the marine environment.

Foraging aquatic mammals must divide their time between obtaining oxygen at the water surface and prey beneath the water surface. Animals that routinely face high fluctuations in oxygen availability or consumption tend to have a general strategy for preventing oxidative damage that involves appropriate constitutive antioxidant capabilities [5]. Myoglobin-rich muscle allows 30 to 50% of O_2_ stores to be concentrated in muscle [2]. During reversible oxygen binding, the oxygenated form of myoglobin or hemoglobin is easily oxidized to the ferric met form in response to the generation of reactive oxygen species (ROS) [6], which are toxic to living organisms. Therefore, diving mammals must cope with higher rates of ROS generation relative to terrestrial mammals due to alterations in apnea/reoxygenation and ischemia-reperfusion processes [5]. Although it has been shown that dolphins possess active non-protein antioxidant molecules to cope with ROS generation [7], these molecules remain to be characterized. Furthermore, cetaceans inhabit an environment where fresh water is not accessible, and the osmotic gradient favors water loss [8], [9]. Although several physiological adaptations for water conservation in cetaceans have been identified, including reduced rate of respiratory water loss [10], the absence of sweat glands [11] and the production of concentrated urine [9], few studies have examined metabolic adaptations to the hyperosmotic environment.

Plasma amino acid concentrations (aminograms) vary depending on various physiological conditions [12], [13], [14]. Many studies have shown that alterations in human aminograms can be caused by diseases. For example, liver failure [15], [16], renal failure [17], cancer [18], [19], diabetes [20], [21], and cardiovascular disorders [22] have been reported to alter aminograms. As liver fibrosis progresses, Fisher's ratio, which is the ratio of branched-chain amino acids to aromatic amino acids, has been shown to be elevated [23], [24], [25]. Some studies have made use of plasma aminograms to distinguish abnormal subjects from healthy subjects and to diagnosis subtypes and stages of diseases by means of data-mining methods [12], [13], [14]. Such methods rely on the principle that plasma amino acid profiles differ depending on the physiological state of an organism. Since amino acids play crucial roles both as building blocks for proteins and as signaling molecules [26], it is reasonable that different physiological conditions result in different amino acid profiles. Furthermore, an underlying network structure has been demonstrated among plasma aminograms [27], [28], and when the physiological condition changes, the homeostatic set point of plasma amino acids will be altered accordingly.

Although the methodology used to describe changes in physiological conditions by analyzing amino acid profiles is established, very few attempts have been made to use these methods to assess the physiological changes that have occurred in marine mammals over the course of evolution. Since some amino acids have endogenous antioxidant effects [29] and some function as blood osmolytes [30], it is likely that the metabolic machinery for amino acids in marine mammals has changed as a result of adaptations to the marine environment. Network analysis is appropriate for demonstrating differences in the amino acid metabolic machinery because it can illustrate complex interrelations among a multitude of free amino acids.

Because Bottlenose dolphins (*Tursiops truncatus*) are exciting marine mammals to observe and are kept in many marine facilities all over the world, the elucidation of their plasma aminograms could provide a useful tool for assessing cetacean health in the future. The purpose of this study was to look for differences in free amino acids between cetaceans and mice by means of data-mining methods and to provide a physiological understanding of cetaceans with regard to free amino acids and adaptations to the marine environment.

## Methods

### Animals and samplings

This study was reviewed and approved by the Ethics Committees of Azabu University and each respective facility. Blood samples collected from wild cetaceans may be affected by multiple factors such as handling stress and diet [31], [32], and hence they are not permitted for use by the Ethics Committees of Azabu University. Therefore, all blood samples in this study were obtained from captive cetaceans. All cetaceans in this study were healthy, as determined by hematology and clinical blood chemistry profiles and behavioral observations performed by keepers and veterinarians who have experience working with cetaceans. A total of 90 blood samples were collected between January 23, 2006 and August 26, 2008 for analysis of plasma amino acid levels. Of these 90 samples, 51 were from bottlenose dolphins (10 males and 41 females), 16 were from pacific white-sided dolphins (*Lagenorhynchus obliquidens*; 14 males and 2 females), 19 were from Risso's dolphins (*Grampus griseus*; 17 males and 2 females), and 4 were from false killer whales (*Pseudorca crassidens*; 1 male and 3 females). The cetaceans were housed in sea pens enclosed by nets (approximately 18,000 m^2^ and 3–6 m in depth) at the Shimoda Floating Aquarium (Shizuoka, Japan) or in pools (approximately 1,000 m^2^ and 3–5 m in depth) at the World Dolphin Resort (Wakayama, Japan), the Taiji Whale Museum (Wakayama, Japan), the Izu-Mito Sea Paradise (Shizuoka, Japan), the Yokohama-Hakkeijima Sea Paradise (Kanagawa, Japan), the Enoshima Aquarium (Kanagawa, Japan), Adventure World (Wakayama, Japan) or the Niigata City Aquarium Marinepia Nihonkai (Niigata, Japan). Blood samples for amino acid analysis were obtained from the tail fluke of each cetacean. Every effort was made to ensure the safety of the cetaceans and personnel. Each cetacean was carefully monitored for signs of discomfort by staff and veterinarians. To avoid the influence of circadian variations and changes in food intake, blood samples were collected during the same time period (08:00–10:00) before feeding, after the cetaceans had fasted overnight.

The C57BL/6J mice were originally purchased from Charles River Japan, Inc. (Yokohama, Japan), and the ICR mice were originally obtained from Japan CLEA Japan Inc. (Yokohama, Japan). Food and water were given *ad libitum*, and all mice were kept at a constant temperature (23±1°C) under a 12-h light: 12-h dark cycle (lights on at 06:00 h). Mice were deprived of food beginning at 18:00 on the day before sampling. All blood samples from mice were collected by cardiac puncture between 08:00 and 10:00 under anesthesia. Eight plasma samples from adult C57BL/6J mice (all male) and five plasma samples from adult ICR mice (2 males and 3 females) were obtained. Heparin was used as an anticoagulant, and samples were immediately centrifuged at 2,000 g for 10 min to separate the plasma. All of the mice were euthanized under deep anesthesia after the experiment.

Urine samples for amino acid analysis were non-invasively and safely collected from the well-trained dolphins by having the dolphins land at the poolside and urinate in response to the trainer's signal. Nine urine samples from three bottlenose dolphins (all female) were collected within 2 hours of 13:00. Mouse urine samples (5 males and 3 females) were obtained from ICR mice by voluntary urination on acrylic boards between 13:00 and 15:00. Samples were immediately centrifuged at 2,000 g for 5 min to remove impurities.

### Determination of plasma and urine amino acids

Two hundred microliters of plasma and urine were each deproteinized with 400 µL of 5% trichloroacetic acid solution and centrifuged at 10,000 rpm for 15 min at 4°C. The supernatants were filtered through regenerated cellulose (Millipore Co., Bedford, MA, USA). The concentrations of free amino acids in each sample were evaluated using an automated L-8800A Amino Acid Analyzer (Hitachi, Tokyo, Japan) according to a previously described method [33], [34], [35], [36]. Urinary amino acid concentrations were corrected against creatinine values.

### Hierarchical cluster analyses

**Figure 1:**
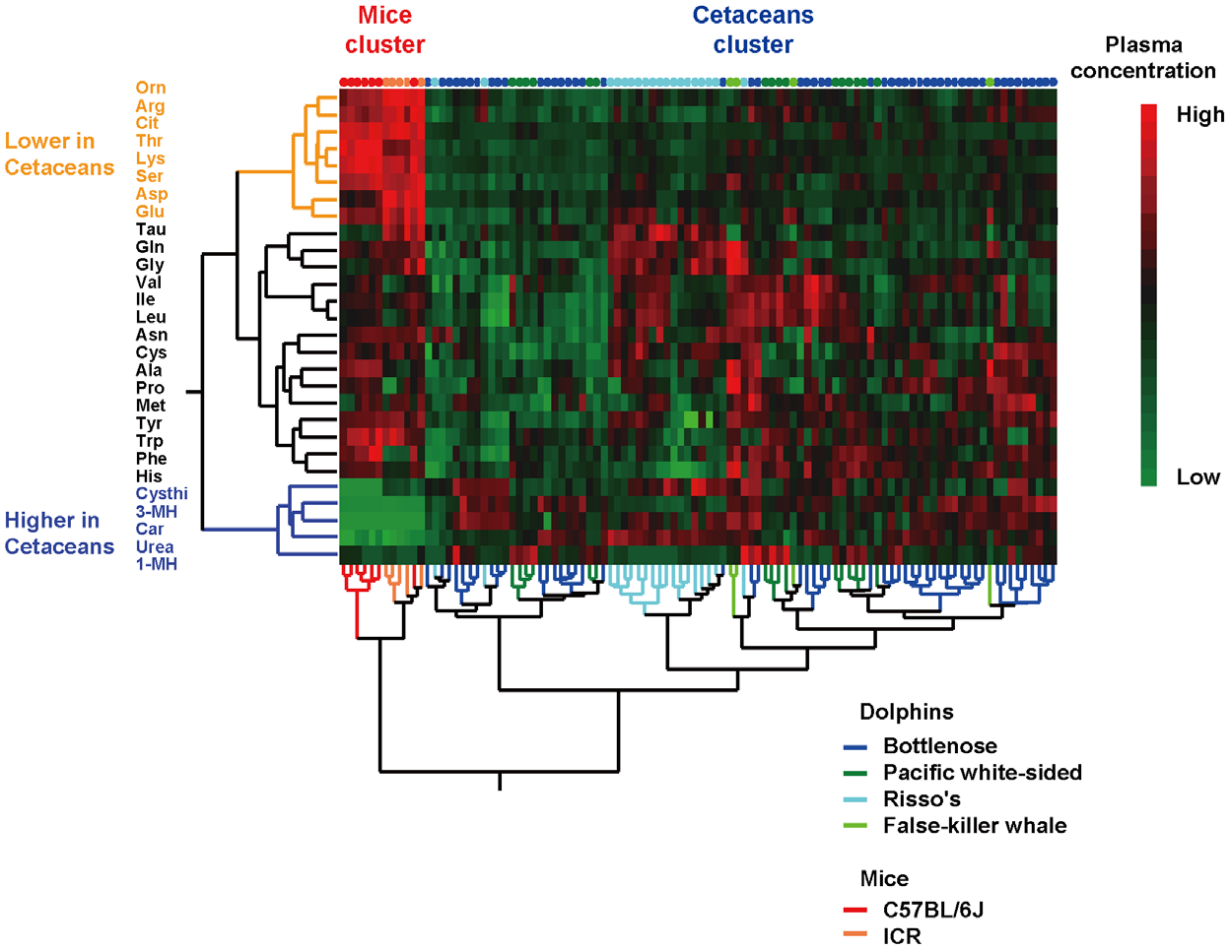
Hierarchical cluster analysis reveals different plasma aminogram patterns in cetaceans and mice. Dendrograms obtained from a hierarchical cluster analysis of the plasma aminograms are shown. Each amino acid concentration was normalized before the analysis, and the red and green squares represent high and low relative concentrations, respectively. Ward's linkage method was performed on the Euclidean distances obtained from the standardized aminogram. Briefly, this method seeks to choose successive clustering steps so as to minimize the increase in the error sum of squares found at each level. Bottlenose dolphins (n = 51), pacific white-sided dolphins (n = 16), Risso's dolphins (n = 19), false-killer whales (n = 4), C57BL/6J mice (n = 8) and ICR mice (n = 5) were used for this analysis. Orn, ornithine; Arg, Arginine; Cit, Citrulline, Thr, threonine; Lys, lysine; Ser, serine; Asp, Aspartate; Glu, Glutamate; Tau, Taurine; Gln, Glutamine; Gly, Glycine; Val, Valine; Ile, Isoleucine; Leu, Leucine; Asn, Asparagine; Cys, Cystine; Ala, Alanine; Pro, Proline; Met, Methionine; Tyr, Tyrosine; Trp, Tryptophan; Phe, Phenylalanine; His, Histidine; Cysthi, Cystathionine; 3-MH. 3-methylhistidine; Car, Carnosine.

Hierarchical cluster analyses were done to obtain a visual representation of the aminograms (Fig. 1). The results are illustrated with a hierarchical tree or dendrogram. Ward's linkage method was performed on Euclidean distances from the standardized aminogram using the JMP 8.0.1 program (SAS Institute Inc., Cary, NC). Briefly, this method uses an analysis of variance (ANOVA) approach to evaluate the distances between clusters and seeks to choose successive clustering steps such that the error sum of squares found at each level is minimized [37].

### Statistical analysis

**Figure 2:**
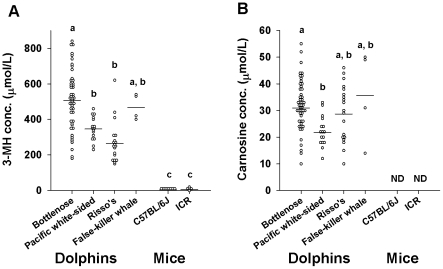
Plasma levels of 3-MH and carnosine are higher in cetaceans. The plasma levels of 3-MH (A) and carnosine (B) are shown. Individual data points are shown as open circles, and bars indicate the average values. The different letters indicate significant differences (*P*<0.05) among groups as determined by Tukey's test. ND  =  not detected. Bottlenose dolphins (n = 51), pacific white-sided dolphins (n = 16), Risso's dolphins (n = 19), false-killer whales (n = 4), C57BL/6J mice (n = 8) and ICR mice (n = 5) were used for this analysis.

**Figure 3:**
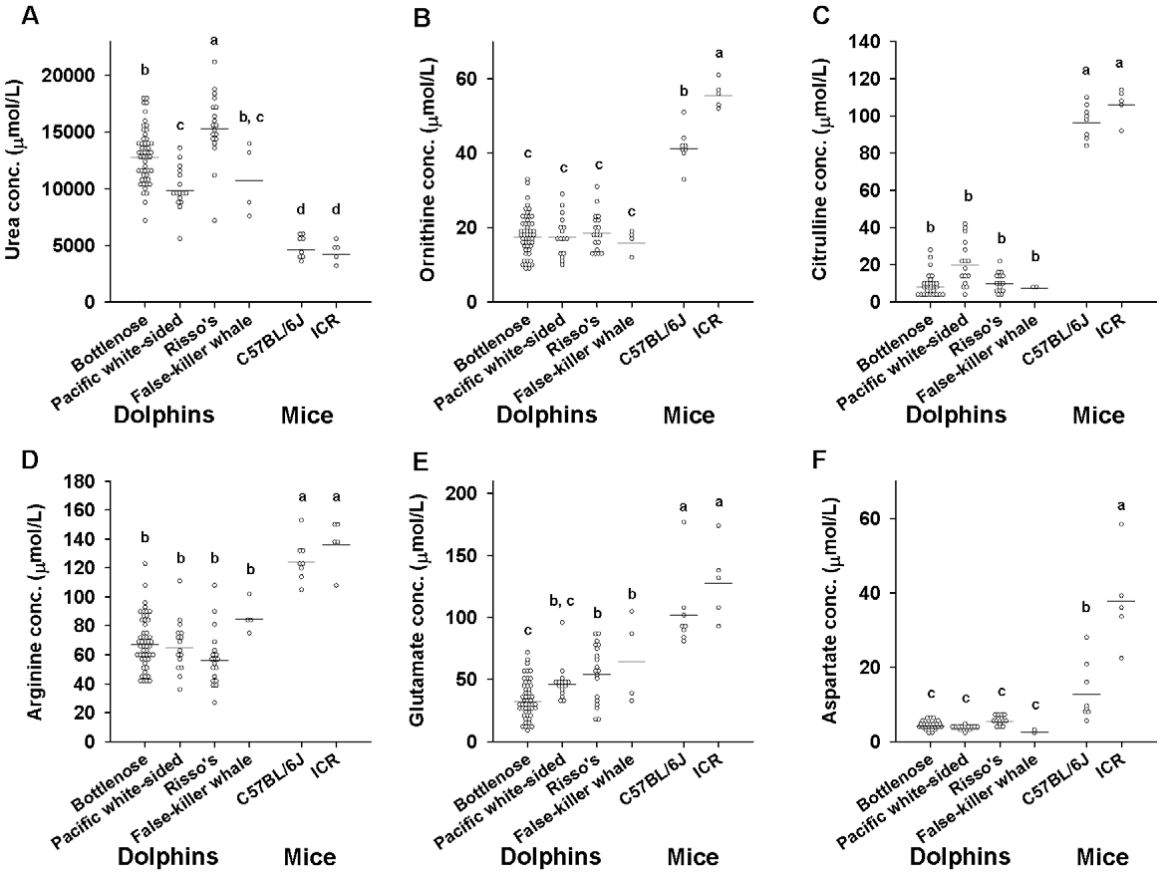
The set point of the urea cycle is different between cetaceans and mice. The plasma levels of urea (A), ornithine (B), citrulline (C), arginine (D), glutamate (E) and aspartate (F) are shown. Individual data points are shown as open circles, and bars indicate the average values. The different letters indicate significant differences (*P<*0.05) among groups as determined using Tukey's test. Bottlenose dolphins (n = 51), pacific white-sided dolphins (n = 16), Risso's dolphins (n = 19), false-killer whales (n = 4), C57BL/6J mice (n = 8) and ICR mice (n = 5) were used for this analysis.

For plasma aminograms, individual data points are expressed as open circles, and bars indicate the average values in Figs. 2 and 3. A one-way ANOVA was used to evaluate the differences between groups. When a significant difference (*P*<0.05) was observed, post-hoc analyses were conducted using Tukey's test. For urine aminograms, the *P*-values were calculated by linear mixed effects models (LME)-associated ANOVA. The LME takes into account correlations between repeated measurements within the same individuals. The LME-associated ANOVAs were considered statistically significant when *P*<0.001 (Table 1).

**Table 1:** Urine concentrations (mean ± SEM µmol/mg creatinine) of amino acids in bottlenose dolphins and mice.

Amino acids	Bottlenose dolphins	Mice
	(n = 9)	(n = 8)
Σ proteinogenic amino acids	1.05±0.16*	3.76±0.22
3-Methylhistidine	0.06±0.01*	0.19±0.03
Carnosine †	37.70±2.89*	‡1.24±0.22

†Levels of carnosine were described as mean ± SEM × 10^−2^ µmol/mg creatinine.

‡Carnosine was only detected in four samples of mouse urine.

*Significantly different between the bottlenose dolphins and mice (*P*<0.001, LME-associated ANOVA).

## Results

### Cluster analyses of plasma aminograms

Fig. 1 shows colored blocks representing the plasma amino acid concentrations of each individual sample. In this representation, red represents a relatively high concentration and green represents a relatively low concentration after normalization. Individual samples and amino acids were ordered based on hierarchical cluster analysis. Since the dendrogram chooses clustering steps that minimize the increase in the error sum of squares at each level, the formation of distinguishable clusters that differ between cetaceans and mice illustrates that the aminogram patterns differ remarkably between these two groups. According to the amino acid cluster formations, levels of ornithine, arginine, citrulline, threonine, lysine, serine, aspartate and glutamate were lower in cetaceans than in mice, whereas levels of cystathionine, 3-methylhistidine (MH), carnosine, urea and 1-MH were higher in cetaceans than in mice.

### Plasma 3-MH and carnosine concentrations

The plasma levels of 3-MH and carnosine are depicted in Figs. 2A and B, respectively. Plasma levels of 3-MH (F = 34.06, *P*<0.001) and carnosine were much higher in cetaceans than in mice. Note that plasma carnosine was not detected in either strain of mice. Anserine which is carnosine-related metabolite, was not detected in either cetaceans or mice.

### Urea cycle-related amino acid levels in plasma

**Figure 4:**
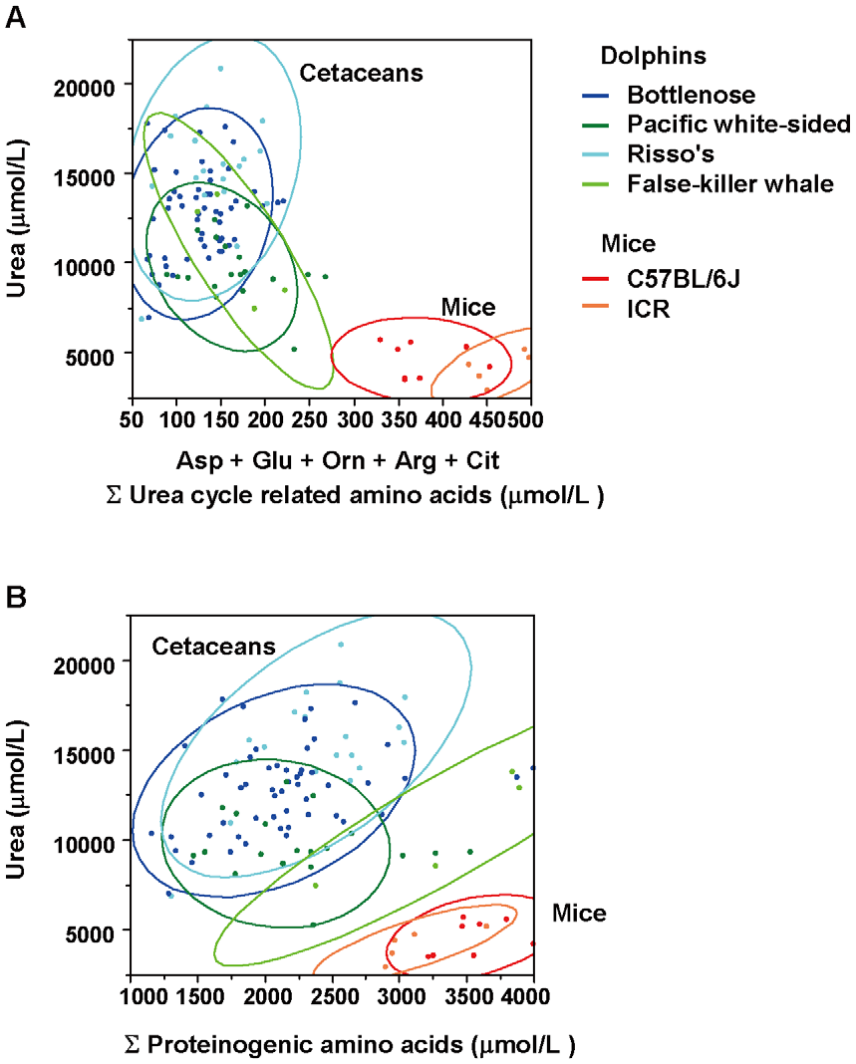
The ratio of urea to urea cycle-related amino acids is different between cetaceans and mice. (A) Plasma urea levels are plotted against plasma levels of Σ urea cycle-related amino acids (i.e., the sum of aspartate, glutamate, ornithine, arginine and citrulline). (B) Plasma urea levels are plotted against plasma levels of Σ proteinogenic amino acids (i.e., the sum of alanine, cysteine, aspartate, glutamate, phenylalanine, glycine, histidine, isoleucine, lysine, leucine, methionine, asparagine, proline, glutamine, arginine, serine, threonine, valine, tryptophan and tyrosine). The colored circles indicate 95% probability ellipses. Bottlenose dolphins (n = 51), pacific white-sided dolphins (n = 16), Risso's dolphins (n = 19), false-killer whales (n = 4), C57BL/6J mice (n = 8) and ICR mice (n = 5) were used for this analysis.

Levels of urea cycle-related amino acids (urea, ornithine, citrulline, arginine, glutamate and aspartate) were assessed in the plasma samples (Fig. 3). Although the levels of urea were two to three times higher in cetaceans than in mice (F = 36.84, *P*<0.001), the levels of ornithine (F = 72.28, *P*<0.001), citrulline (F = 341.41, *P*<0.001), arginine (F = 31.53, *P*<0.001), glutamate (F  = 35.03, *P*<0.001) and aspartate (F = 82.04, *P*<0.001) were significantly lower in cetaceans. Fig. 4 shows scatterplots of the plasma amino acid concentrations of individual samples. Correlation plots of Σ urea cycle-related amino acids versus urea in plasma are depicted in Fig. 4A, and Σ proteinogenic amino acids versus urea are depicted in Fig. 4B. While weak positive linear correlations were found between Σ proteinogenic amino acids and urea within each group (Fig. 4B), no correlations were found between Σ urea cycle-related amino acids and urea (Fig. 4A). The colored circles, which show 95% probability ellipses, did not overlap between cetaceans and mice.

### Urinary amino acid levels

The concentrations of amino acids in the urine samples were expressed as µmol per mg of creatinine (Table 1) as opposed to µmol per 24 hours, which is typically used and preferred in analogous human studies. We chose these units because it was impossible to perform 24-hour collections of urine samples from bottlenose dolphins on a routine basis.

The urinary concentrations of Σ proteinogenic amino acids in dolphins were significantly lower than those in mice (F = 100.99, *P*<0.001). The concentrations of 3-MH in the urine of the dolphins and mice were 0.05±0.01 and 0.19±0.03 µmol/mg creatinine, respectively; this difference was significant (F = 21.25, *P*<0.001). The concentration of carnosine in the urine of the dolphins was approximately 30 times higher than that of mice (F = 85.89, *P*<0.001).

Here, we employed creatinine, which is an end-product of creatine energy metabolism and excreted by kidney, to correct urinary amino acid levels. Attention must be paid, however, since energy metabolism and kidney function in marine mammals may be different from those in terrestrial mammals, and creatine is produced from arginine, which was reduced in cetaceans. As far as 3-MH and carnosine levels, since the correction by creatinine value did not greatly affect the result of 3-MH and carnosine levels whose concentrations were greatly differed between cetaceans and mice, these alterations would be due to physiological differences and not due to any artifacts. The standardization methods of urine amino acid levels in marine mammals should be consolidated in future.

## Discussion

To our knowledge, this is the first study to comprehensively analyze and compare plasma amino acid concentrations in multiple cetaceans. Comparisons of amino acid profiles will lead to a better understanding of the physiological role of their biochemical effects, and such effects may allow marine mammals to live entirely underwater. These data may also be useful indicators for assessing cetacean health conditions in the future. Since the aim of this study was to unveil the characteristic amino acid metabolism observed in cetaceans, compared with representative terrestrial animals, we chose mice as one of the most intensively investigated terrestrial model animals. Mice and rats are known to have similar amino acid metabolism, and both of them are frequently used as model animals to investigate amino acid metabolism [12], [13], [35], [36], [38].

Plasma amino acids can be viewed as a network that adapts to various physiological conditions and that may become perturbed in response to disease and/or physiological insults [12], [13], [14], [27], [28]. In addition to the metabolic pathways within cells, there are various levels of networks within the body; one such level is represented by the transport of substrates such as amino acids between organ systems via the bloodstream. Our results (Fig. 1) suggest that cluster analysis can be used to visualize a multitude of amino acid relationships among different species, which can help us understand the complex interrelations underlying metabolic adaptations to the environment. As shown in Fig. 1, aminograms of bottlenose dolphins, pacific white-sided dolphins, Risso's dolphins and false-killer whales formed a cetacean cluster that was markedly different from the cluster obtained from C57BL/6J and ICR mice, indicating that the physiological states of the cetaceans and the mice were markedly different. Among the 28 amino acids that we examined in this study, characteristic differences between the two clusters were found for ornithine, arginine, citrulline, threonine, lysine, serine, aspartate and glutamate (which were lower in cetaceans), and cystathionine, 3-MH, carnosine, urea and 1-MH (which were increased in cetaceans).

The most distinctive differences between cetaceans and mice were found in the concentrations of plasma 3-MH and carnosine (Fig. 2). Plasma levels of 3-MH in the cetaceans were 50 to 100 times greater than those in the terrestrial mammals. Most 3-MH is formed by the post-translational methylation of specific histidine residues in the myofibrillar proteins actin and myosin, and 3-MH cannot be reused for protein synthesis. In many terrestrial mammals, 3-MH does not undergo catabolism and is primarily excreted in the urine [39]. However, since the urinary 3-MH levels of cetaceans were significantly lower than those of terrestrial mammals (Table 1) [40], [41], whereas plasma levels were significantly higher, it is likely that cetaceans reabsorb 3-MH. Some animals such as pigs, which have balenine in their skeletal muscle, reabsorb 3-MH for synthesizing balenine [41]. Cetaceans might also reabsorb 3-MH for synthesis of balenine, and the further analyses whether balenine level in the cetacean skeletal muscle were upregulated or not will provide the physiological meaning of 3-MH reabsorption. At least, 3-MH is known to function as an endogenous antioxidant [29], and thus, these high concentrations of 3-MH in the plasma appear to play an important role in cetacean physiology. Although carnosine has not been detected in human [42] or mouse plasma (Fig. 2), it was enriched in cetacean plasma. Also, carnosine is a naturally occurring antioxidant [29] and transition metal ion-sequestering agent. It has been shown to act as an anti-glycating agent as well, inhibiting the formation of advanced glycation end products. Through its distinctive combination of antioxidant and anti-glycating properties, carnosine can attenuate cellular oxidative stress and inhibit the intracellular formation of ROS and reactive nitrogen species. As noted by Reddy et al. [43], “by controlling oxidative stress, suppressing glycation, and chelating metal ions, carnosine is able to reduce harmful sequelae such as DNA damage.” This has been supported by several studies [29], [43], [44], [45], [46], [47]. Carnosine and carnosine-related antioxidants have therefore recently attracted much attention as possible therapeutic agents for humans [47], [48]. Since anserine, which is carnosine-related metabolite, was not detected, it was likely that the metabolic system to uniquely upregulate carnosine level exist in cetaceans. Because diving mammals must cope with high rates of ROS generation due to alterations in apnea/reoxygenation and ischemia-reperfusion processes [5], high concentrations of these amino acids in the plasma could play an important antioxidant role and would be beneficial for aquatic life. Higher levels of urine carnosine in cetaceans than in mice (Table 1) suggest the synthesis rate of carnosine might be enhanced in marine mammals.

Because characteristic differences were also found in urea and urea cycle-related amino acids, we performed a correlation analysis focusing on urea cycle-related amino acids (Figs. 3 and 4). The urea cycle plays a major role in the generation of urea using amino acids derived from either dietary proteins or endogenous origins via conversion of deamination-derived ammonia in the liver. In the urea cycle, amino acids enter the pathway for urea synthesis via the transdeamination or transamination routes. In either route, α-ketoglutarate accepts an amino group from the donor amino acid to form glutamate. In the former pathway, glutamate is deaminated to form α-ketoglutarate and ammonium ions, and then the ammonium is incorporated into carbamoyl phosphate, which in turn reacts with ornithine to enter the urea cycle as citrulline. In the latter pathway, oxaloacetate accepts an amino group from glutamate to form aspartate. This aspartate now carries a second amino group into the urea cycle by condensing with citrulline to form argininosuccinate. Argininosuccinate is then cleaved to form fumarate and arginine. Finally, arginine is hydrolyzed to ornithine and urea [49]. As shown in Figs. 3 and 4A, cetacean plasma levels of aspartate, glutamate, ornithine, arginine and citrulline, which are utilized to synthesize urea via the urea cycle, were much lower than those of mice, whereas the level of plasma urea itself was much higher in cetaceans. When all the proteinogenic amino acids are compared (Fig. 4B), their plot distributions are seen to be less divergent between mice and cetaceans than those of the urea cycle-related amino acids (Fig. 4A). This indicates that, in cetaceans, the urea and urea cycle-related amino acids are at unique homeostatic set points. Urea functions as a major blood osmolyte [30], and in some animals, blood osmotic pressure is primarily elevated through the retention of urea [50]. For example, plasma urea levels are high in sharks; in a hyperosmotic environment, this adaptation prevents the loss of body water [50]. It is possible that maintenance of a higher level of plasma urea, i.e., by converting urea cycle-related amino acids to urea by adjusting the set point of the urea cycle, would be advantageous for marine mammals. Such a metabolic adaptation might facilitate body water conservation in the hyperosmotic sea water environment.

Whereas our study provides the levels of amino acids in the plasma and urine of various cetaceans, variations in plasma amino acid levels due to species, sex, age, season, living situation and health have not yet been determined. Hematology, clinical blood chemistry profiles and behavioral indices are routinely used in marine mammals and in many terrestrial mammals to monitor health status. However, Waples and Gales [51] reported a case of fatality of a bottlenose dolphin without associated changes in hematology or clinical blood chemistry profiles, suggesting that other physiological tests should be incorporated into the management techniques. Because plasma aminograms are used to distinguish abnormal subjects from healthy subjects in humans [12], [13], [14], it is likely that accumulated plasma and/or urine aminogram data will provide useful indicators for the assessment of cetacean health in artificial environments.

In conclusion, our results indicate that cetaceans and terrestrial mammals have different metabolic machinery for amino acids that facilitates adaptation to the ocean environment. To fully understand the metabolic adaptations of marine mammals required for a lifetime spent entirely underwater, further research is necessary regarding the distribution of amino acids in other marine mammals, such as *Pinnipedia*, which live both underwater and on land. The evolutionary closer terrestrial animals such as *Artiodactyla* will also be needed to be investigated in future. Network analyses of plasma aminograms in those animals will shed light on the origins of the unique homeostatic regulation of 3-MH, carnosine, urea and urea cycle-related amino acid levels observed in cetaceans.
